# The Emergence of Urban Land Use Patterns Driven by Dispersion and Aggregation Mechanisms

**DOI:** 10.1371/journal.pone.0080309

**Published:** 2013-12-27

**Authors:** James Decraene, Christopher Monterola, Gary Kee Khoon Lee, Terence Gih Guang Hung, Michael Batty

**Affiliations:** 1 Complex Systems Group, Computing Science Department, Institute of High Performance Computing, Agency for Science Technology and Research, Singapore, Singapore; 2 Centre for Advanced Spatial Analysis, University College London, London, United Kingdom; University of Namur, Belgium

## Abstract

We employ a cellular-automata to reconstruct the land use patterns of cities that we characterize by two measures of spatial heterogeneity: (a) a variant of *spatial entropy*, which measures the spread of residential, business, and industrial activity sectors, and (b) an *index of dissimilarity*, which quantifies the degree of spatial mixing of these land use activity parcels. A minimalist and bottom-up approach is adopted that utilizes a limited set of three parameters which represent the forces which determine the extent to which each of these sectors spatially aggregate into clusters. The dispersion degrees of the land uses are governed by a fixed pre-specified power-law distribution based on empirical observations in other cities. Our method is then used to reconstruct land use patterns for the city state of Singapore and a selection of North American cities. We demonstrate the emergence of land use patterns that exhibit comparable visual features to the actual city maps defining our case studies whilst sharing similar spatial characteristics. Our work provides a complementary approach to other measures of urban spatial structure that differentiate cities by their land use patterns resulting from bottom-up dispersion and aggregation processes.

## Introduction

To address the challenges posed by rapid urbanization, ageing populations, rising real energy costs, and environmental constraints, among others, methods to quantify the similarities and differences between existing land allocations among cities are essential. Such measures may allow comparisons between the quality of life and general well-being of the resident population, hence, bringing us a step closer to achieving an objective metric for properly representing sustainability indices [1]. Defining a measure to differentiate cities remains a critical challenge both for domain practitioners and information scientists. We previously demonstrated [2] that by combining the relative mixing of distinct entities (business, residential, industrial sectors) in a given area and their relative spread over the entire locality, we are able to quantify the degree of similarity and distinctiveness of land use design attributes among cities. In particular, we previously demonstrated a procedure using a measure of spatial entropy and an index of dissimilarity to capture the distinctness of the forces reflecting dispersion and aggregation between the different land use types. We showed that the combination of these two measures provide a straightforward means of probing the common and unique attributes of Singapore and other North American cities. For example, we report that a distinctive spatial characteristic of cities is for industrial districts to be consistently clustered and segregated while residential are generally dispersed and mixed with business areas thus confirming this long standing observation for many developed cities. While this measure has also been utilized in other research [3]–[5] in addition to other indexes which account for accessibility, population density, residential property value and passenger trip volumes, these studies are limited to single cities. In contrast, here we use these measures as a basis to differentiate many cities according to their land use spatial properties.

In this paper, we build from our previous work and investigate the factors contributing to the emergence urban land use patterns based on a dynamics that results from dispersion and segregation mechanisms. A dynamical urban growth model is proposed that aims to reconstruct artificial cities from the bottom-up with specific spatial entropy and dissimilarity index values. This is a cellular automata model that relies essentially on the ranges of influence of the different land use sectors. These ranges determine the aggregation tendencies of each sector which are diffused according to power-law distribution (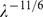) throughout the city extent, the exponent of which we take from Bettencourt's analysis of its possible range of values [6].

## Methods

We first describe the assumptions driving the development of the cellular automata (CA) model that we use. The iterative growth procedure is then detailed. Finally, the methods used to compute the spatial entropy and dissimilarity index are presented. We note that the term spatial entropy is used in several different ways in the literature with its use here associated with the definition of entropy states as pertaining to land use activities distributed across the city space. A long line of such spatial entropy measures are indicated in [7]–[10]. The assumptions adopted to drive the development of the model are as follows: The central business district (CBD) is located at the original settlement site of the city (as the CBD is typically located at or near the origin of the market place in the city). The probability of developing a plot depends on its value and the land value decreases rapidly, following a power law distribution, with respect to the distance to the CBD [11]. Note that this is a general assumption which ignores local specific factors (e.g. the presence of particular amenities or sites such as marinas, cemeteries or heritage sites) which may increase or decrease the land value locally. Moreover, the land value is the principal factor which determines the density and type (i.e. business, residential and industrial) of the land development, where business, followed by residential uses are more likely to be developed than industries in high value land plots. This type of land use type distribution with respect to land values can be regarded as a simplication of the Von Thunen model [12] first generalized to urban systems by Alonso [13]. When such a development is created or expanding, its initial or incremental size varies. Finally, residential buildings are not likely to be developed within an area occupied or adjacent to industries, as the latter would decrease the land plot value for potential residential purposes.

**Figure 1:**
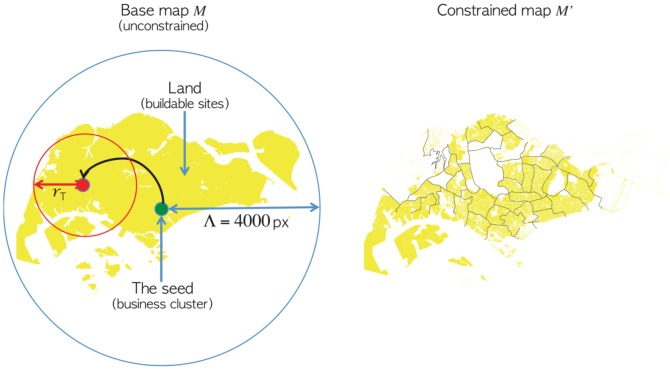
The Singapore model. Highlighted in the right figure are the areas that remains (yellow color) after non-developable lands are removed.

Motivated by the above assumptions, a CA is proposed where the cells, i.e., a land plot unit -a pixel -of 64 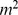 of a given sector are injected into a base city map, see Fig. 1 for the example of Singapore, at each iteration until all land use cells (that is, total number extracted from the actual city land use zoning map) are allocated and placed. The model is initialized with a single business seed located at the likely original site of settlement which constitutes the CBD. The dispersion of residential and business cells is governed by a power law probability density function (Eq. 1) where the probability of developing these activities decays rapidly as the distance to the CBD increases:
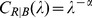
(1)with 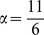 and 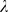 is the distance from the CBD. 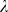 ranges from 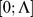 where 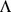 is the distance between the CBD and the farthest location within the city boundaries. The exponent value is taken from Bettencourt's analysis [6] but it is also consistent with the values of power law exponents that arise in studies of the fractality of cities [14]. This power law distribution reflects the rapid decay in the land value radiating from the CBD. The probability of development for industrial buildings often follows a reversed trend where the probability increases rapidly as the distance to the CBD increases, i.e. the exponent becomes positive, and this is the assumption we use here. Further details on the effects of the spatial resolution upon the computation of both the spatial entropy and dissimilarity index can be found in [2]. Note that in this paper, series of experiments were conducted to evaluate and identify a sufficient and suitable spatial resolution to measure spatial entropy and to best discriminate land use plots according to their dissimilarities; Those experimental outcomes were utilized to set a satisfactory spatial resolution here, i.e., 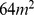 per pixel.

Following this random dispersion process, a cell of type 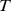 in {R, B, I} (where 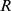 = Residential, 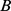 = Business, 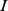 = Industrial), is placed at some location on the two-dimensional base map. Then, the cell aggregates with other cells of the same type found (if any) in the area whose radius is defined by the range of influence 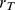, a distance measured in pixels where each pixel is of actual length 8 meters. In this model, only three parameters are identified and varied in our study to reconstruct the city land use patterns where the ranges of influence for each land use sector are defined as 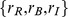 for the residential, business and industrial sectors respectively. These parameters determine the aggregation levels of the land use sectors: The higher 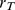, the more clustered the land use cells will be. Note that the ranges of influence also affect the dispersion degree to some extent at the local level.

A relatively small range of influence would tend to produce more dispersed developments in the area delimited by the range of influence. Nevertheless, this dispersion degree due to the range of influence may be mitigated by the growth of multiple clusters which may eventually merge with each others. The growth iteration loop is now detailed.

### The growth iteration loop


**Figure 2:**
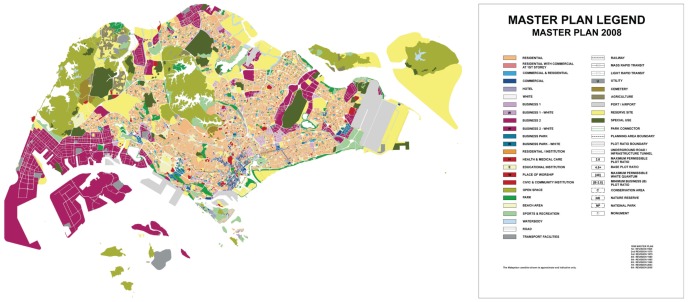
The Singapore master plan. The actual land use map depicted in Fig. 3 was extracted from the URA ((Urban Redevelopment Authority)) Singapore master plan 2008 where the residential, business and industrial land use sectors are the aggregations of relevant sub-categories. See Appendix S1 for details and data sources.

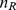
, 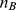 and 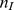 are set as the numbers of residential, business and industrial pixels found in the actual city map, see Fig. 2 where 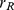, 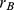, 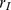 are set manually. Parameter screening experiments over the ranges of influence are reported later. A map location or pixel (with an actual length equal to 8 meters) is said to be “developable” if it is land (i.e. not a water area) and not already occupied one of the land use sector activities. Constrained models rely on simplified city maps 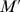, see Fig. 1, where forests, distribution centres, parks, reserved lands and roads are not considered as developable land plots. When a cluster is “grown” or created with a given size 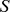 of cells, the new cells are placed at the periphery of the cluster at random with the distance to cluster equal to rand(4), i.e. 4×8 = 32 actual meters. This signifies that when new developments are built, these would not be constructed immediately right next to another one but rather in the area that we assume here to be within 4 pixels or an average of 32 meters in actual units. If no space is available, in other words if the cluster is completely surrounded by other developments, then the given cluster is not expanded further.

- **Initialization** :
**Set** 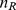, 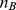, 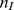, 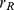, 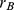, 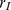

**Set** CBD location at the city's original place of settlement
**Set** maximum jump distance 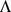 equal to the distance between the CBD location and farthest developable land location in the map 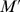

**Set** maximum cluster cell incremental size 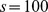

**Create** seed cell cluster of type 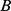 with size 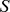 = rand(s) at CBD location; decrement 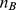 by 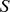

- **Repeat** until 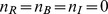 :
  1. **Select** available, i.e. 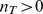, cell type 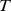 at random
  2. **Set** random location 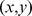 where the Euclidean distance from the CBD is a random variable drawn from 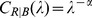 **if** 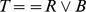, with 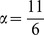 and 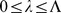. When 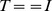 the exponent is positive.
  3. **If** land plot at location 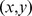 is developable **then** Look up for closest cluster of cells 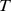 in area centered at 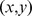 with radius 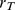:
**Set** 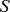 = rand(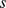)
**If** a cluster is found **then** make it grow by size 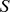

**Else**, create a new cluster 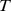 of size 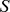 at location 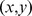

    Decrement 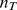 by 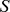

  4. **If** 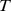 =  = I and its location is adjacent to a cluster of type 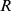 then remove cluster of cells 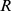 and increment 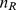 accordingly.

In the next section, we will detail the procedure for computing spatial entropy and the dissimilarity index.

### Spatial entropy and the dissimilarity index

We now present the procedure for computing the relative spatial entropy 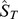 and dissimilarity index 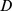 of different land types. We first consider a land use zoning map 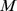 as a regular grid composed of 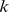 frames. Each frame is itself constituted of 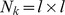 pixels. As the zoning maps are colour-coded by land use type, a pixel is similarly categorized by its colour value. Given a land use type 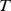, the density of pixels of type 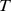 per frame is denoted by 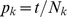 where 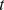 is the number of pixels of type 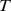 found in the frame. This measure of spatial entropy evaluated per frame is utilized to examine the dispersion of land use types in cities. For a land use type 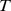, the entropy is given by:
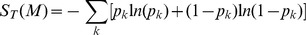
and is normalized as:
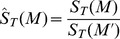
where 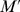 is a copy of 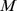 with same density of pixels 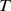 but with the latter being randomly distributed (using a uniform distribution) over the city land. We have conducted sensitivity analysis (which is not reported here) to examine the variance in the resulting spatial entropy measurements and these results indicate that the variance was negligible since differences were only noted at the 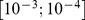 order using sets of 10 independent spatial entropy computations with unique seeds, different zoning maps and frame length values. When 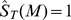, pixels 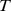 are dispersed throughout all the frames, whereas when 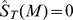, this indicates that pixels 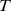 constitute a single cluster.

We then employ the dissimilarity index to characterize the degree of aggregation/segregation [15] between land use types. Given two land use types 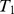 and 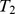, the dissimilarity index is expressed as:
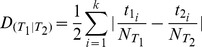
with 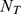 being the total number of pixels 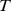 found in the zoning map. The limit 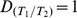 implies that the land uses of types 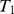 and 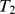 are fully segregated whereas 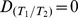 indicates an even distribution of both land use types throughout the frames.

The computation of both indexes depends on the length 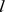 utilized to define the frames. We previously demonstrated that a suitable length frame to compute entropy is 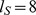 meters whereas 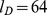 meters for the computation of the dissimilarity index.

## Results and Discussion

We first report and detail our results for the city state of Singapore. We then apply our CA approach to a selection of North American cities, whose simulation outcomes are used for our comparative analysis.

### Emergence of the land use patterns in Singapore

**Figure 3:**
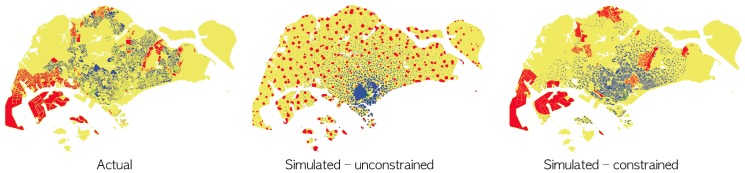
The Singapore land use patterns. Actual versus simulated city maps with and without compartmental constraints. Blue, green and red coloured pixels correspond to residential, business and industrial areas respectively. The actual map of Singapore was adapted from the Singapore Urban Redevelopment Authority master plan 2008 in which we discarded land use categories that are not directly related to the residential, business and industrial sectors.

Fig. 3 depicts two illustrative simulation outcomes compared with the actual land use of Singapore. The unconstrained model does not include any land constraints where distribution centers, reserved lands, forests, parks, roads and military sites are available for development in contrast to the constrained model which considers these constraints and thus limits the areas available for residential, business and industrial development. The unconstrained model can be regarded as a single growth container with no inner sub-compartments. On the other hand, the constrained model devises sub-compartments which may physically limit the growth of the land use sectors.

Tables 1 and 2 list the actual spatial entropy and dissimilarity values computed for the city state of Singapore. We note that residential and business areas are equivalently dispersed throughout the frames (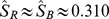). In contrast, the entropy 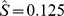 of industrial areas indicates that industries are relatively more clustered. The residential/business coupling displays the lowest degree of segregation. In contrast, the business/industrial and industrial/residential couplings are relatively more segregated. The results indicate the tendency of the residential and business communities to be situated far from the industrial sites and vice-versa.

**Table 1:** Simulation results summary - spatial entropy.

		Simulated values - 〈*Ŝ*〉			Actual values - *Ŝ*		
City		*R*	*B*	*I*	*R*	*B*	*I*
2*Singapore	unconstrained	0.295±0.006	0.307±0.007	0.097±0.001	2*0.290	2*0.336	2*0.125
	constrained	0.281±0.003	0.310±0.006	0.112±0.011			
Houston		0.616±0.002	0.264±0.002	0.157±0.002	0.504	0.277	0.177
Las Vegas		0.393±0.001	0.239±0.003	0.201±0.013	0.467	0.225	0.221
New York		0.526±0.001	0.339±0.003	0.272±0.002	0.663	0.408	0.316
San Francisco		0.532±0.003	0.309±0.006	0.264±0.019	0.606	0.403	0.232
Seattle		0.440±0.004	0.222±0.003	0.161±0.010	0.493	0.279	0.141
Toronto		0.405±0.002	0.330±0.003	0.173±0.006	0.348	0.259	0.137
Vancouver		0.505±0.002	0.279±0.003	0.191±0.017	0.668	0.373	0.214
Washington DC		0.511±0.006	0.306±0.004	0.195±0.027	0.506	0.252	0.174

The mean and standard deviation values were computed over 10 individual repeat simulation runs using unique seeds. Results for all cities are based using the compartmental constrained models, except for Singapore land use which was also reconstructed using the unconstrained approach. Note that the standard deviations of the different trial measurements in the average is 2.67% of the mean value (0.74%, 1.37%, 5.89% for 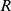, 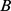, 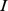 respectively), indicative of the robustness of the evolved patterns.

**Table 2:** Simulation results summary - dissimilarity index.

		Simulated values - 〈*D*〉			Actual values - *D*		
City		*R*|*B*	*B*|*I*	*R*|*I*	*R*|*B*	*B*|*I*	*R*|*I*
2*Singapore	unconstrained	0.805±0.008	0.945±0.006	0.931±0.004	2*0.807	2*0.986	2*0.976
	constrained	0.758±0.014	0.933±0.012	0.980±0.003			
Houston		0.678±0.005	0.948±0.007	0.935±0.003	0.722	0.804	0.902
Las Vegas		0.740±0.009	0.970±0.011	0.948±0.010	0.801	0.949	0.995
New York		0.628±0.007	0.894±0.006	0.803±0.005	0.570	0.772	0.833
San Francisco		0.744±0.022	0.962±0.021	0.943±0.007	0.791	0.904	0.910
Seattle		0.700±0.014	0.925±0.015	0.951±0.004	0.775	0.925	0.966
Toronto		0.788±0.005	0.940±0.007	0.968±0.002	0.854	0.976	0.964
Vancouver		0.574±0.016	0.965±0.012	0.955±0.006	0.765	0.761	0.925
Washington DC		0.633±0.018	0.973±0.011	0.960±0.010	0.863	0.910	0.921

The mean and standard deviation values were computed over 10 individual repeat simulation runs using unique seeds. Results for all cities are based using the compartmental constrained models, except for Singapore land use which was also reconstructed using the unconstrained approach. Note that the standard deviations of the different trial measurements in the average is 1.14% of the mean value (1.71%, 1.14%, 0.58% for 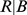, 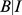, 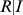 respectively), indicative of the robustness of the evolved patterns.

Table 1 and 2 also summarize the entropy and dissimilarity results obtained using the best-fit models using the constrained and unconstrained maps. To obtain these results a series of parameter screening experiments where conducted. Example parameter screening results can be found in Appendix S1.

In Table 3, we observe that both models exhibit comparable fitting results with 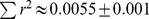. Nevertheless when comparing maps resulting from these illustrative simulation runs, depicted in Fig. 3, it can be observed that despite all maps sharing similar quantitative spatial measures, their appearance differs widely. Specifically, the unconstrained model compares poorly with the actual map of Singapore. On the other hand, when comparing the actual map of Singapore and the map based on the constrained land, we observe a stronger visual resemblance.

**Table 3:** Simulation fitting results summary.

		Fitted parameters			Error	Base
City		*r_R_*	*r_B_*	*r_I_*	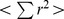	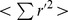
2*Singapore	unconstrained	50	175	250	0.005±0.001	3.905±0.004
	constrained	50	100	1250	0.007±0.001	3.166±0.003
Houston		5	150	400	0.038±0.002	3.576±0.001
Las Vegas		5	150	500	0.013±0.001	3.149±0.005
New York		5	80	200	0.045±0.002	2.092±0.002
San Francisco		5	100	600	0.024±0.003	2.833±0.005
Seattle		5	100	1100	0.013±0.001	3.487±0.003
Toronto		30	130	1100	0.016±0.001	3.494±0.002
Vancouver		2	50	1000	0.118±0.008	2.720±0.006
Washington DC		5	100	1000	0.065±0.007	3.257±0.004

The error column reports the sum of squared residuals over the spatial entropy for each land use type and dissimilarity indexes between the actual city map and best-fit reconstructed maps. The base column details the fitted results using the constrained models and a random uniform distribution of the land use sectors throughout the city maps. Thus the base column reports controlled experimental results which highlight the differences when no dispersion and aggregation mechanisms are implemented. The above result shows that the model reported is three orders of magnitude statistically more accurate than a random growth model.

**Figure 4:**
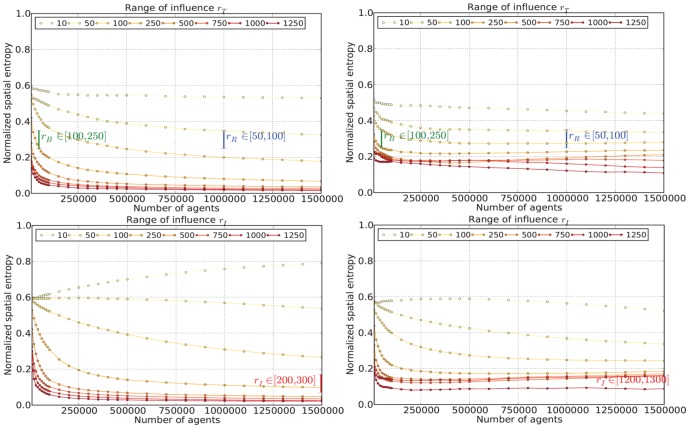
Parameter screening for the Singapore model for the range of influence for residential/business and industrial areas (Left column: unconstrained; Right column: constrained). Preliminary experiments were conducted to identify potentially suitable range values (highlighted in coloured text in the figures) for the respective ranges of influence for the three land use sectors (residential - blue, business - green and industrial - red) for the city state of Singapore. These experiments assisted in limiting the search space to find the best-fit parameters to match the actual spatial entropy values given the specific number of land use cells (pixels), i.e 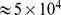, 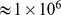 and 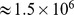 for business, residential and industrial pixels respectively in Singapore. Similar experiments were conducted for our selection of North American cities but these are not reported here.

**Figure 5:**
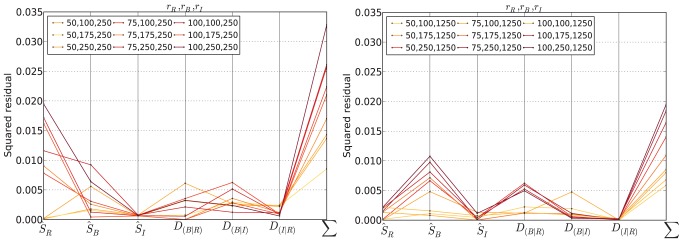
Parallel coordinate plot of squared residuals for a limited set of model parameter configurations for the Singapore model (Left: unconstrained; Right: constrained). Using the range values determined in preliminary experiments (Fig. 4), we conducted a limited set of experiments to find the best-fit model parameters. Each column reports the squared residual for each target spatial entropy and dissimilarity indexes values extracted from the actual land use map of Singapore. From these sets of experiments, we identify the sets 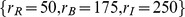 and 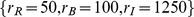 as the best-fit parameters for the unconstrained and constrained models respectively. Similarly, further experiments were conducted to identify the best-fit model parameters for the remaining cities.

When examining the best-fit parameters (Figs. 4 and 5), we first note that both models share the same range of influence for residential areas with 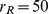 an actual distance of 400 meters. The constrained model exhibits a higher 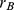 than the unconstrained with a difference 75 pixels or 600 meters. In contrast, the industrial ranges of influence are consistently one order of magnitude higher or a difference of 1000 pixels or 8000 meters. This relatively large 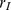 value suggests that the compartmental constraints impact heavily on areas available or suitable, when considering the land value, for industrial purposes in Singapore: The scarce availability of suitable areas for industrial purposes is here compensated by a significantly higher 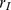 which lead to the formation of larger industrial clusters. Nevertheless these clusters are fragmented locally due to the presence of transport infrastructures (i.e. roads). Whereas in the unconstrained model, we observe an almost homogeneous distribution of tightly clustered and medium sized industrial developments.

**Figure 6:**
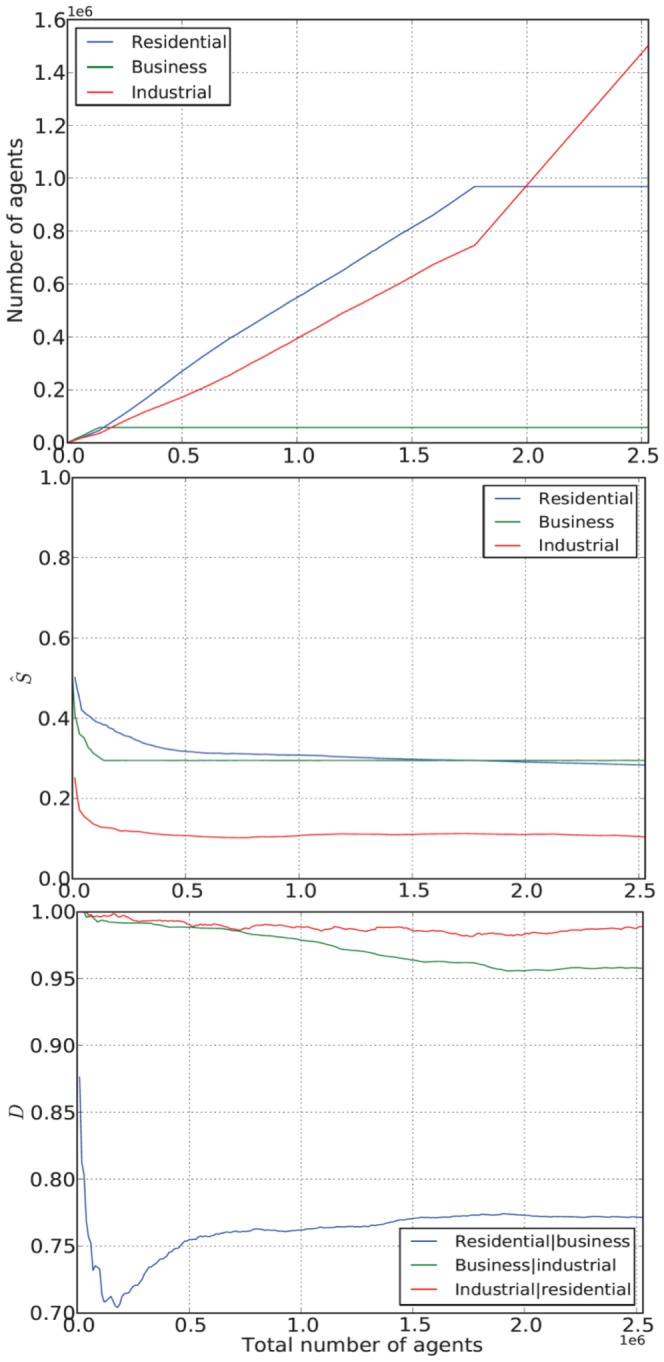
Dynamics of spatial entropy and dissimilarity index against number of cells. These results were extracted from a single illustrative simulation run.

Fig. 6 shows the dynamics of 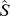 entropy and 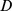 dissimilarity measures against the number of cells created during the iterative growth process using the constrained model. It can be noted that the spatial entropy stabilizes (with 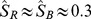 and 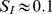) when the total number of created cells reaches half a million. Little fluctuations in 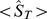 over the next two million created cells can be observed whereas the dynamics of the dissimilarity index differs where 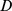 reaches equilibrium after the growth of at least two million cells. This indicates that the dispersion process settles first when compared with the aggregation process which continues to vary to some extent as new cells are created.

### Reconstructing and differentiating urban land use patterns

**Figure 7:**
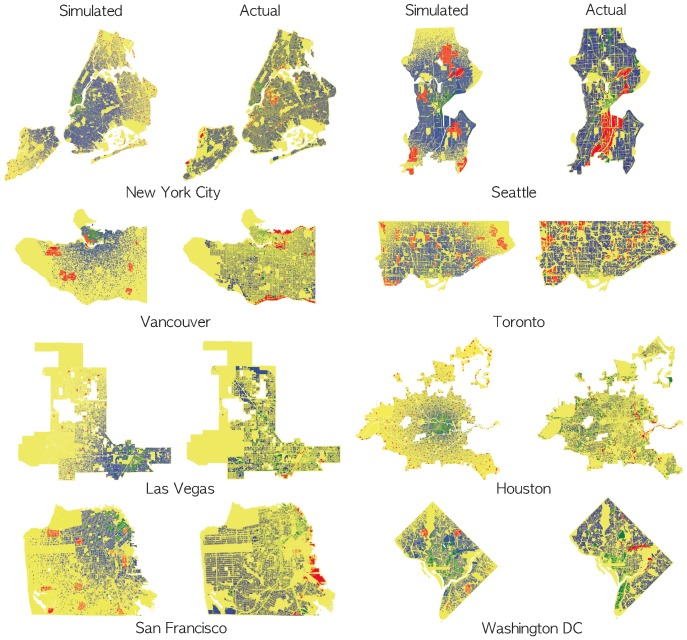
Reconstructed versus actual city land use maps. Simulated figures is randomly chosen representative sample of 10 runs whose statistical resemblance to actual land use is reported in Tables 1–3.

We now consider applications of the model to eight representative US and Canadian cities with populations all over 500,000 so that we might make comparisons with Singapore. Fig. 7 illustrates the best-fit reconstructed land use maps for the selected American cities. We observe that the visual resemblance differs with Seattle, Toronto and Washington DC presenting, from a qualitative point of view, the best resemblance with the actual land use maps. We note that these cities tend to be more structured along the lines of cities that have developed with less sprawl than the other cities in the set although topographic considerations are also important as in the case of New York City.

A common difference can be observed in the remaining cities where a gradient in residential density is noted in the reconstructed land use patterns; whereas a relatively more homogeneous distribution of residential building is observable in the actual land use maps. This gradient pattern results from the land value power law distribution function used in the model which leads to the concentration of residential areas around the city cores. Although this currently depreciates the visual resemblance between reconstructed and actual land use patterns, we hypothesize that this issue is due the two-dimensional nature of the land use maps. When considering the elevation of buildings, it is possible that we may retrieve this observed gradient in residential density where high-rise buildings are likely to be found around the CBD, whereas low-density residential areas would be located in the outer city areas. This may merit further investigation in future work.

Moreover, when examining the actual land use maps of Houston and Las Vegas, we note that multiple business clusters exist with no single CBD clearly noticeable, and this accords to our impression that these cities are much more recent in origin, developing very rapidly during the last half century when the CBD has been in decline in many North American cities. This contrasts with the reconstructed land use map where a relatively large CBD can be observed. Moreover, we note that for all other land use maps, including both reconstructed and actual ones, a principal CBD can clearly be distinguished. In the actual map of Las Vegas, we note the presence of secondary large business developments situated along Rancho Drive which connects the CBD to the north-western part of the city with this particular feature observable in the reconstructed map of Las Vegas. With regard to Houston, we may consider the relatively large area where potentially, multiple business clusters can be observed due to the presence of multiple cities, each of which, would exhibit a distinct business core. In the current model, a single seed, i.e. the CBD was introduced in the model initialization. Utilizing multiple seeds is expected to improve the accuracy of the results, but for purpose of simplicity and to illustrate the general validity of our method, we restrict our discussion to a single source initialization. Finally, note that Houston is the only city from our selection which does not follow a planned zoning map whose potential implications remain to be investigated further.

**Figure 8:**
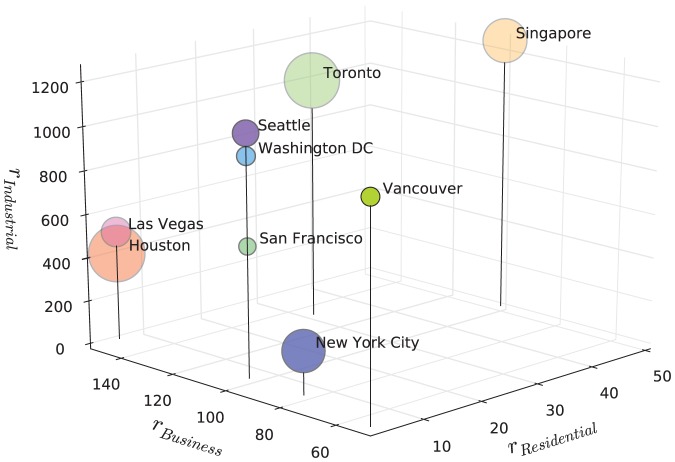
Cities differentiated by ranges of influence. The bubble size denotes the total land area of the city.

Figure 8 depicts the cities differentiated by their best-fit ranges of influence. No clear correlations can be identified between the total city areas and ranges of influence. This is rather counter-intuitive as we expected the range of influence for industrial developments to increase as the land area increases, indeed all cities exhibit low spatial entropy for industrial areas with highly clustered industrial development clearly observable. As industries tend to be located in the periphery of these cities, if the city area increases, then we might naively expect the industrial range of influence to increase accordingly to maintain the formation of large industrial clusters. Nevertheless, we observe that for some large cities such as New York City or Houston, the range of influence is relatively low varying from 200 to 400 pixels (1600 to 3200 actual meters). In contrast, smaller cities, namely Seattle, Washington DC, Vancouver and San Francisco present higher ranges of influence for industries ranging from 500 to 1000 pixels. We also note that the range of aggregation level for business areas (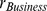) is generally twice that of residential (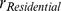). On the other hand, range of industrial areas (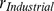) is an order of magnitude higher than both 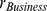) and 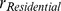. This numerical finding is consistent with our previous claims that industrial sectors are more significantly clustered compare to residential and business establishments.

Quite clearly from Fig. 8, the newer cities of Las Vegas and Houston which are cities of the automobile age with hardly any public transport, display a bigger spread of business activities than the older east coast cities of New York and the Canadian cities of Vancouver and Toronto. This mix also shows a mild correlation with size in terms of land area but one problem that we have with all this analysis (and this is generic to the field), is that city size definitions are highly variable and the maps that we have sourced to develop this work are not chosen with the best boundary definitions in mind, largely because we have no control over these issues. However what we do find is that in a planned city-state such as Singapore, the ranges over which activities segregate or cluster are much larger than those for more organically growing cities. Planning control is thus likely to destroy natural patterns as implied by comparison of these measures in Singapore with the North American cities. This suggests that we should develop further research along comparative lines which deals with different political, cultural and developmental regimes with respect to such urban patterns.

In conclusion, we have reported a procedure for simulating the emergence of land use patterns observed in Singapore and other North American cities. Using cellular automata, we have shown that we can evolve visually comparable spatial patterns of cities by growing the actual geographical areas using: 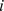). *diffusion* and *aggregation* dynamics, and 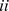). imposing actual *land use constraints* (i.e. defining non-developable areas). Simultaneous recovery of six independently measured attributes (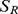, 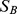, 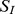, 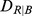, 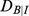, 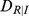) from only three parameters (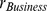, 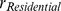, 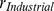) hints at the robustness and statistical accuracy of our paradigm. These define our directions for future work but we are optimistic that this approach will yield simple growth models that define patterns of different urban activities that can be traced to a small, simple and parsimonious set of key physical parameters.

## Supporting Information

Appendix S1 **Sources of maps.**
(PDF)

Movie S1 **Simulation for the emergence of land use allocation of Singapore using diffusion and aggregation mechanisms.**
(MOV)
