# Supplementary material for: The Emergence of Urban Land Use Patterns Driven by Dispersion and Aggregation Mechanisms
Source: PLoS One. 2013 Dec 27;8(12):e80309. doi: 10.1371/journal.pone.0080309 (PMC3873928; doi:10.1371/journal.pone.0080309)
Supplement: Appendix S1 — Sources of maps. (PDF) [file pone.0080309.s001.pdf]

Appendix: The emergence of urban land use patterns driven by dispersion and aggregation mechanisms

The source code and utilized data are publicly available in this URL: `https://drive.google.com/?usp=chrome_app#folders/0B8h3D2o57F37NENINTFsRjhndDA`.

Listed below are the sources of the different maps (raster images). As the maps were devised by different institutions, the land use type classification differed. As we only considered three land use types, i.e. residential, business and industrial, we aggregated the relevant (sub-)land use types accordingly within these three categories.

Houston

Source	Houston-Galveston council, 2008.
Url	`http://mapbook.h-gac.com/land_use_land_cover.htm`
Residential	Residential
Business	Commercial
Industrial	Industrial, Utility, ROW

Las Vegas

Source	City of Las Vegas, Planning & Development Dept, 2012.
Url	`http://www.lasvegasnevada.gov/Publications/maps.htm`
Residential	Residence Estates, Single-Family Residential Restricted ,Single Family Residential, Mobile/Manufactured Home Residence, Medium-Low Density Residential, Medium Density Residential, High Density Residential, Apartment
Business	Professional Offices And Parking, Professional Office, Office, Designed Commercial, Limited Commercial, General Commercial, Commercial Industrial
Industrial	Industrial

New York City

Source	New York City, Department of City Planning, 2010.
Url	`http://www.nyc.gov/html/dcp/html/landusefacts/landusefactsmaps.shtml`
Residential	Residential
Business	Commercial
Industrial	Industrial / Manufacturing

San Francisco

Source	San Francisco, Planning Department, 2012.
Url	`http://www.sf-planning.org/index.aspx?page=1569`
Residential	Residential, House Character Districts, Mixed (Houses & Apartments) Districts, Residential-Commercial Combined Districts, Residential Transit Oriented Districts, Downtown Residential Districts, Chinatown Mixed Use Districts (Residential), South of Market Mixed Use Districts (Residential Enclave, Residential/Service), Eastern Neighborhoods Mixed Use Districts (Mixed Use, Residential)
Business	Neighborhood Commercial Districts, Neighborhood Commercial Transit Districts, Chinatown Mixed Use Districts (Commercial, Visitor Retail, Community Business), Commercial Districts, Eastern Neighborhoods Mixed Use Districts (Mixed Use, Office)
Industrial	Industrial Districts

Seattle

Source	City of Seattle, Department of Planning and Development, 2012.
Url	`http://www.seattle.gov/dpd/Research/Zoning_Maps/default.asp`
Residential	Single Family 5000, Single Family 7200, Single Family 9600, Residential Small Lot, Lowrise, Midrise, Highrise
Business	Neighborhood Commercial, Commercial, Downtown Office Core, Downtown Harborfront, Downtown Mixed, International District, Pike Market Mixed
Industrial	Industrial Commercial, Industrial General 1, Industrial General 2

Singapore

Source	Singapore Urban Redevelopment Authority, Master Plan, 2008.
Url	`http://www.ura.gov.sg/mp08/map.jsf?goToRegion=SIN`
Residential	Residential, Residential with commercial at First Storey
Business	Commercial and residential, Business 1, Business 1 white, Business Park, Business Park white
Industrial	Business 2, Business 2 white

Toronto

Source	Toronto City Planning , 2012.
Url	`http://www.toronto.ca/zoning/city-wide_maps.htm`
Residential	Residential, Residential Apartment
Business	Commercial
Industrial	Employment Industrial

Vancouver

Source	City of Vancouver, adapted from BC assessment data, 2001.
Url	`http://vancouver.ca/commsvcs/planning/stats/landuse/pdf/landuse2001.pdf`
Residential	Single family dwelling, Duplex, Rowhouses, or Multiple Conservation Dwelling, Apartment, Apartment and Commercial
Business	Commercial
Industrial	Industrial or Utilities

Washington D.C.

Source	Government of the District of Columbia, Office of Planning 2012.
Url	`http://planning.dc.gov/DC/Planning/DC+Data+and+Maps/Map+Library/Comprehensive+Plan+Future+Land+Use`
Residential	Low/Moderate/Medium/High Density Residential
Business	Low/Moderate/Medium/High Density Commercial
Industrial	Production, Distribution, and Repair
